# Preparation of GST Inhibitor Nanoparticle Drug Delivery System and Its Reversal Effect on the Multidrug Resistance in Oral Carcinoma

**DOI:** 10.3390/nano5041571

**Published:** 2015-09-29

**Authors:** Bing Han, Yanli Wang, Lan Wang, Zuhui Shang, Shuang Wang, Jin Pei

**Affiliations:** 1 School of Pharmaceutical Sciences, Jilin University, No.1266 Fujin Road, Changchun 130012, China; E-Mail: hanb@jlu.edu.cn; 2 Changchun Women and Children Health Hospital, No. 962 Xinfa Road, Changchun 130061, China; E-Mail: ccwangyanli@163.com; 3 School of Pharmaceutical Engineering, Shenyang Pharmaceutical University, No.103 Wenhua Road, Shenyang 110016, China; E-Mails: 322hb@sohu.com (L.W.); shangzuhui@hotmail.com (Z.S.); 4 China Japan Union Hospital, Jilin University, No. 126 Xiantai Street, Changchun 130033, China; E-Mail: ccwangshuang@163.com

**Keywords:** nanoparticle, GST inhibitor, redox-sensitive, self-assembly

## Abstract

During the chemotherapy of cancer, drug resistance is the first issue that chemotherapeutic drugs cannot be effectively used for the treatment of cancers repeatedly for a long term, and the main reason for this is that tumor cell detoxification is mediated by GSH (glutathione) catalyzed by GST (glutathione-S-transferase). In this study, a GST inhibitor, ethacrynic acid (ECA), was designed to be coupled with methoxy poly(ethylene glycol)-poly(lactide) (MPEG–PLA) by disulfide bonds to prepare methoxy poly(ethylene glycol)-poly(lactide)-disulphide bond-mthacrynic acid (MPEG–PLA–SS–ECA) as a carrier material of the nanoparticles. Nanoparticles of pingyangmycin (PYM) and carboplatin (CBP) were prepared, respectively, and their physicochemical properties were investigated. The ECA at the disulfide could be released in the presence of GSH, the pingyangmycin, carboplatin and ECA were all uniformly released, and the nanoparticles could release all the drugs completely within 10 days. The half maximal inhibitory concentration (IC_50_) of the prepared MPEG–PLA–SS–ECA/CBP and MPEG–PLA–SS–ECA/PYM nanoparticles in drug-resistant oral squamous cell carcinoma cell lines SCC15/CBP and SCC15/PYM cells was 12.68 μg·mL^−1^ and 12.76 μg·mL^−1^, respectively; the resistant factor RF of them in the drug-resistant cells were 1.51 and 1.24, respectively, indicating that MPEG–PLA–SS–ECA nanoparticles can reverse the drug resistance of these two drug-resistant cells.

## 1. Introduction

Malignant tumors are still the primary diseases to seriously threat human health and life today. Studies have shown that the formation of tumors is a constantly cumulative process involving more factors, and more stages. Chemotherapy remains the most primary means for the treatment of tumor so far, so that the individual difference in the therapeutic effect of chemotherapeutic drugs is significant since there are great differences in chemotherapeutic drugs used for the treatment of tumors. The medication targeting each different individual tumor, based on the existing unified clinical experience, easily leads to the primary resistance of tumor cells to chemotherapeutic drugs [1]. The development of drug-induced multidrug resistance (MDR) of tumor tissues during the course of chemotherapy seriously affects the therapeutic effect of tumors. It is difficult for the resistance of tumors to be reversed once it develops, which means a failure of the treatment, and furthermore the development of multidrug resistance may indicate a cross resistance of tumor cells that are never exposed to other antitumor drugs, resulting in the ineffectiveness of tumor treatment in patients with tumors [2,3]. Oral squamous cell carcinoma is one of the common malignant tumors in the head and neck, its mortality is ranked sixth of all tumors, and chemotherapy has played an important role in the treatment of oral cancer, including the preoperative induction chemotherapy, postoperative chemotherapy, palliative chemotherapy for advanced tumor, *etc*. It is particularly common for oral squamous carcinoma cells to develop resistance, leading to failure in chemotherapy, which may be the main cause for the recrudescence of oral squamous cell carcinoma [4,5,6,7]. Due to the affection of various factors, such as types of the chemotherapeutic drugs, tumor types, tumor differentiation stages, and drug targets, administration ways, and interactions between the drugs and their targets, the drug-resistant phenotypes of tumor cells are not the same, and *in vivo* and *in vitro* studies have found that the mechanism of multidrug resistance in oral cancer is a complicated process in which a variety of factors are involved, such as related protein expression, enzyme mediation, gene mutation, and expression deletion of stem cell targets [6,8,9,10]. Currently, studies both at home and abroad, focus primarily on the following aspects: the decrease in the intake of drugs by cells, increase in the efflux of drugs by cells, change in the cell apoptosis-related pathway, change in the drug targeting molecules, enhanced DNA repair mechanism and increase in activities of the drug metabolic enzymes in tumor cells, of which glutathionine-S-transferase (GST) that can catalyze glutathione to bind chemotherapeutic drugs to form different complexes that can be transported out of the cells as the substrates of MRP1, one of the most important multidrug resistance genes [11,12,13,14,15]. The involving of GSH in the multidrug resistance in cancer cells is currently believed to be related to multidrug resistance-associated protein (MRP) and glutathione-S-transferase. GSH (glutathione), a naturally synthesized peptide in the cytoplasm of human beings is composed of glutamic acid, cysteine and glycine, contains thiol, and is an important component of intracellular antioxidant system, and, moreover, it can bind to many electrophilic drugs and poisons as a major intracellular electron donor. GST itself can bind to many lipophilic drugs, thus promoting the detoxification and efflux of anticancer drugs. Therefore, the inhibition of GST activities is a more significant and direct way to reduce the multidrug resistance of tumors. In addition, there is a significant difference in the redox potential between inside and outside cells, showing a partial oxidation outside cells and a partial reduction inside cells [13,14,16]. GSH/(GSSG) is the main redox couple in animal cells and makes the cells have antioxidant capacity. The content of intracellular GSH is 2–3 orders of magnitudes higher than that of GSH in extracellular fluid [17,18]. In addition, it has been found that the intracellular concentration of GSH in cancer cells is generally elevated, several times higher than those in normal cells [19]. Based on these different points, redox-sensitive polymers, biological connectors and nano-carrier containing disulfide bonds have attracted much public attention in many fields in the past few years.

**Scheme 1:**
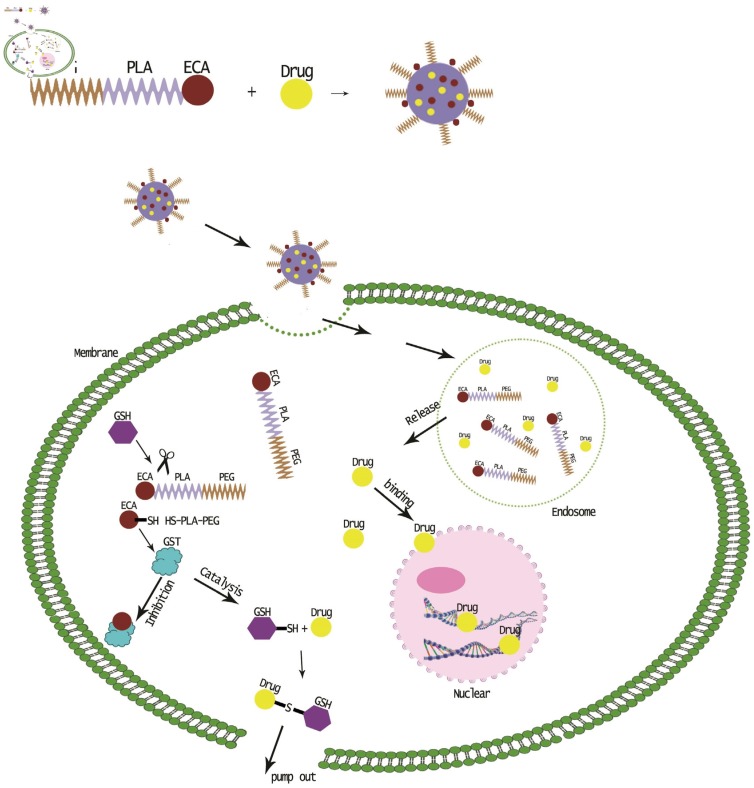
Reversal effect of glutathionine-S-transferase (GST) inhibitor nanoparticle drug delivery system on the multidrug resistance in cancer cells.

In this study, ethacrynic acid (ECA), a GST inhibitor, was coupled with poly (ethylene glycol)-poly (lactic acid) (MPEG–PLA) through disulfide bonds to prepare a redox-sensitive polymer MPEG–PLA–SS–ECA, and the polymer could self-assemble for the preparation of nanoparticles. Both the solubility and stability of the drug in the form of the prepared nanoparticles could be increased by the modification of the polyethylene glycol on the surface of the nanoparticles. The duration of the drug with a small particle size in the body and the concentration time of it at the tumor site could be ensured to be lengthened; that is, enhanced permeability and retention effect (EPR) [20,21]. By introducing the disulfide bond that could be cleaved by cysteine, the targeted release of GST inhibitors can be achieved to reverse the multidrug resistance in tumor cells. Human oral squamous cell carcinoma SCC15 cells were used for the study, in which SCC15/PYM and SCC15/CBP cell strains resistant to the chemotherapeutic drugs pingyangmycin (bleomycin) and carboplatin commonly used for oral squamous cell carcinoma were induced, respectively, and the pingyangmycin and carboplatin were encapsuled with MPEG–PLA–SS–ECA to prepare MPEG–PLA–SS–ECA/PYM and MPEG–PLA–SS–ECA/CBP nanoparticles, respectively. The prepared nanoparticles could accumulate in the tumor cells to effectively reduce the expression of GST protein in tumor cells and reverse the resistance of the drug-resistant cell strains (Scheme 1).

## 2. Results and Discussion

### 2.1. Synthesis and Characterization of MPEG–PLA–SS–ECA Polymer

**Figure 1:**
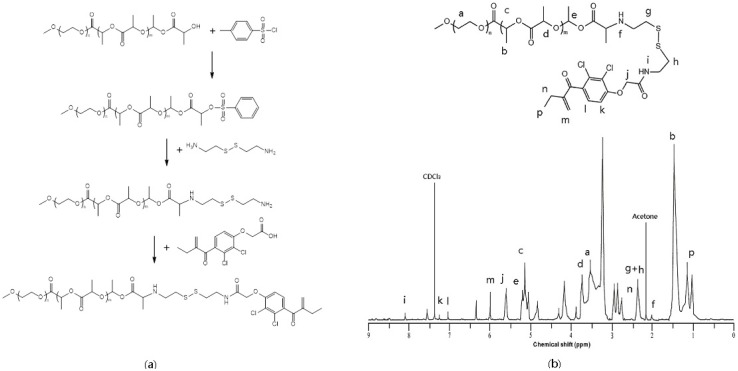
(**a**) Synthesis of the polymer conjugates of ethacrynic acid (ECA); (**b**) ^1^H NMR (Proton Nuclear Magnetic Resonance Test) spectra of polymer conjugate ECA inCDCl_3_.

The chemical structure of MPEG–PLA–SS–ECA was identified based on the nuclear magnetic resonance spectrum. AECA-grafted polymer carrier material was designed and synthesized experimentally, and the ECA in the carrier was linked to the hydrophobic end of amphiphilic block copolymer MPEG–PLA through disulfide bonds. This carrier material was used as the polymer material to prepare the nanoparticles, and the PEG on its surface may ensure the nanoparticles to retain their EPR and enable them to reach to the focus as soon as possible through the venous blood [21,22,23]. The ECA with disulfide bonds designed in this study was aimed at the dissociation of ECA in the highly expressed GSH environment in tumor cells to make the ECA exert its effect as a GST inhibitor to reduce the multidrug resistance of tumor cells [24,25]. Due to more active groups of the ECA, the synthetic reaction at the ECA end should be minimized as far as possible. Therefore, MPEG–PLA–NH–CH2–CH2–SS–CH2–CH2–NH2 was primarily synthesized and only one step reaction was needed for the reaction of it with the ECA to obtain the end product (Figure 1).

### 2.2. Preparation and Related Physicochemical Properties of MPEG–PLA–SS–ECA Nanoparticles

**Figure 2:**
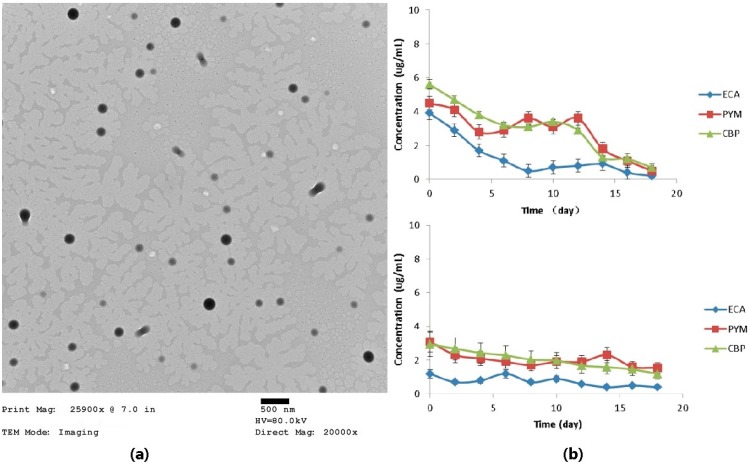
(**a**) Transmission electron microscope (TEM) pictures of MPEG–PLA–SS–ECA nanoparticles; (**b**) Cumulative release rates of nanoparticles *in vitro*.

As shown in the transmission electron microscope (TEM) images (Figure 2), the surface of MPEG–PLA–SS–ECA nanoparticles was relatively smooth, the shape was regular, the size was uniform, and they were not aggregated with each other. The *in vitro* drug release was conducted by dialysis in PBS buffer system and PBS solution containing 10 mM GSH, respectively, in which the 10 mM concentration was used to simulate the environment inside the tumor cells [26,27]. The drug release was measured in both environments, respectively, and meanwhile the release of coupled ethacrynic acid was also inspected. Compared with the common preparations of pingyangmycin and carboplatin, the initial exorbitant release was not seen in the different groups of nanoparticles in which the drugs were encapsuled under the condition of PBS buffer, suggesting that the nanoparticles with disulfide bonds should be relatively stable in the normal cellular environment. During eight days in the environment of 10 mM GSH, 70% of the drugs in nanoparticles prepared with MPEG–PLA–SS–ECA as the material could be released, and the release of drugs in the nanoparticles in the single PBS buffer was less than 25%, indicating that the nanoparticles could rapidly release the drugs only inside cells, but not easily release them in other body fluids (lower concentrations of GSH). The particle size of nanoparticles prepared with MPEG–PLA–SS–ECA was about 200 nm, and there was a PEG structure on their surface so that it could be judged that MPEG–PLA–SS–ECA nanoparticles should have EPR. Thus, We could expect that when the MPEG–PLA–SS–ECA nanoparticles were injected into the body, their EPR could be exerted to make the nanoparticles accumulate in tumor tissues, and when they were swallowed by tumor cells through endocytosis, the degradation of polymer PLA and at the same time the release of drug should be accelerated in cells due to the unique GSH concentration sensitivity, the disconnection of disulfide bonds, and the protonated effect of sulfydryl groups on the surface of nanoparticles [28,29]. There, leased compounds containing the structure of ethacrynic acid could inhibit the activity of GST and lower the multidrug resistance of cells. The release period of pingyangmycin and carboplatin nanoparticles was 10 days and the release was relatively uniform. The release period of loaded drugs was relatively consistent with that of the ECA, indicating that ECA could counteract the cell detoxification of GST induced by pingyangmycin and carboplatin when they exert their effects in the tumor cells.

### 2.3. Identification of the Induced Drug-Resistant SCC15/CBP and SCC15/PYM Cells

**Figure 3:**
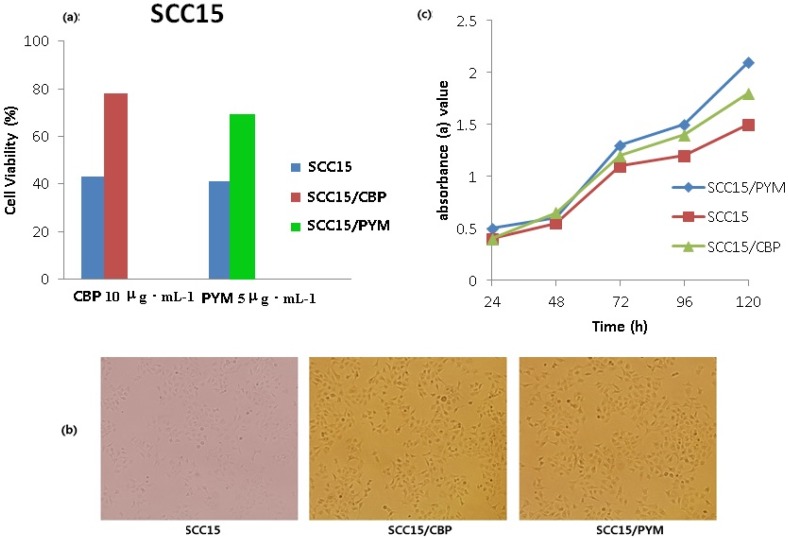
(**a**)The survival rate of drug resistant cells at drug resistant concentration for 72 h; (**b**) P-gp expression of drug resistant cells; and (**c**) the growth curve of SCC15, SCC15/CBP, SCC15/PYM cells.

Figure 3a shows a significant difference in the survival rate between drug-resistant cells induced at the drug resistance mass concentration for 72 h and the parental cells (*p* < 0.05). The positively colored parts of cells expressing P glycoprotein (P-gp) were in the cytoplasm and the cell membrane, and gathered in and around cells, and the positively expressed rate of drug-resistant cells was significantly higher than that of the parental cells (Figure 3b), indicating that the drug-resistance of SCC15/CBP and SCC15/PYM cell strains should be developed. After the successive *in vitro* induction and culture of the cells for 24 weeks, SCC15/CBP- and SCC15/PYM-resistant cell lines were established. Final drug resistance mass concentrations of carboplatin and pingyangmycin were 10 µg·mL^−1^ and 5 μg·mL^−1^, respectively. The growth of drug-resistant cells was not significantly affected after the development of their resistance, and the proliferation speed of drug-resistant cells was faster than that of the parent cell, but there was no significant difference between them (*p* > 0.05, Figure 3c).

### 2.4. Endocytosis of MPEG–PLA–SS–ECA Nanoparticles in Drug-Resistant Oral Squamous Cell Carcinoma Cells

**Figure 4:**
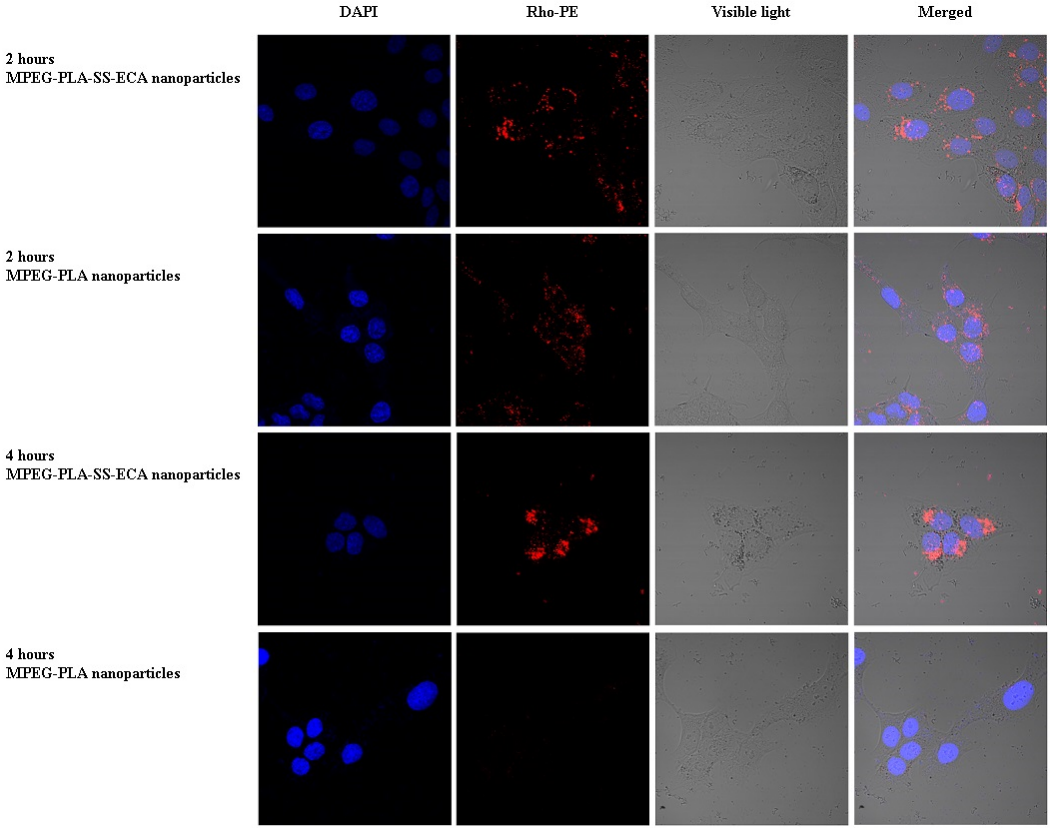
Cellular uptake behavior of MPEG–PLA–SS–ECA nanoparticles.

*In vitro* endocytosis of nanoparticles in each group was investigated. In accordance with the nanoparticle preparation method described above, rhodamine was encapsuled in nanoparticles to prepare MPEG–PLA–SS–ECA/rhodamine and MPEG–PLA/rhodamine, and then the prepared MPEG–PLA–SS–ECA/rhodamine and MPEG–PLA/rhodamine nanoparticles were incubated with the drug-resistant oral squamous cell carcinoma cells SCC15/PYM for 4 h, in which the encapsuled rhodamine was used as the labeled agent and a confocal microscope was used for the observation and recording at the different time points. The confocal images showed that a large number of MPEG–PLA–SS–ECA and MPEG–PLA nanoparticles labeled with a red accumulated in SCC15/PYM cells, the nanoparticles moved from outside the cells to inside the cells within 0.5–1 h, and all of them could entered the cells 2 h later (Figure 4). MPEG–PLA–SS–ECA nanoparticles can accumulate in the SCC15/PYM cells in 4 h, but MPEG–PLA nanoparticles cannot stay inside the SCC15/PYM cells. It is believed that because GSH and GST in the tumor cells can be induced to attack disulfide bonds of the MPEG–PLA–SS–ECA nanoparticles, the disulfide bonds should be broken up, so that the release of nanoparticles can be accelerated and their water-solubility may be reduced, leading to the retention of nanoparticles in the cytoplasm; with the consumption and the inhibition of GST isoenzymes by etacrynic acid, oral squamous carcinoma cells gradually lose their exocytosis so that MPEG–PLA–SS–ECA nanoparticles can accumulate in the tumor cells.

### 2.5. Effects of MPEG–PLA–SS–ECA on MDR of the Two Drug-Resistant Cells

**Figure 5:**
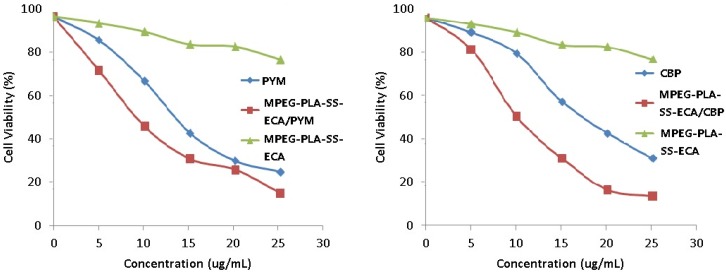
(**a**) SCC15/PYM cells were treated with PYM and MPEG–PLA–SS–ECA/PYM, cell viability and IC_50_ were determined by MTT assay. (**b**) SCC15/CBP cells were treated with CBP and MPEG–PLA–SS–ECA/CBP, Cell viability and IC_50_ were determined by MTT assay.

MPEG–PLA–SS–ECA drug-loaded nanoparticles (loading carboplatin and pingyangmycin) were incubated with SCC15/CBP and SCC15/PYM cells, respectively. MPEG–PLA–SS–ECA/PYM and MPEG–PLA–SS–ECA/CBP were prepared into a solution of 10 μg·mL^−1^ nanoparticles of them, and the solutions were put in the media containing drug-resistant oral squamous cell carcinoma cells SCC15/CBP and SCC15/PYM, respectively. Based on the results of MTT assay, the curves were drawn, and the linear regression equations of the two cell strains were determined, respectively (*p* < 0.05). The calculated results showed that before MPEG–PLA–SS–ECA was used, IC_50_ values of SCC15/CBP and SCC15/PYM were 19.12 μg·mL^−1^ and 15.85 μg·mL^−1^ (Figure 5), respectively, and after MPEG–PLA–SS–ECA was used, those of SCC15/CBP and SCC15/PYM IC50 were 12.68 μg·mL^−1^ and 12.76 μg·mL^−1^, respectively; then RF (resistance fraction) values of SCC15/CBP and SCC15/PYM cells to MPEG–PLA–SS–ECA were 1.51 and 1.24, respectively, indicating that MPEG–PLA–SS–ECA nanoparticles can partially reverse the drug resistance of these two drug-resistant cells.

### 2.6. Inhibition of MPEG–PLA–SS–ECA on GST-π

**Figure 6:**
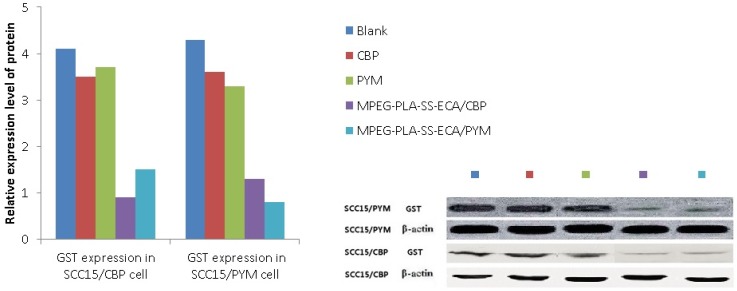
Expression levels of GST protein in drug resistant cells.

The Western blot test results of proteins showed that after the incubation with the drug-loaded nanoparticles for 24 h, the expression of Glutathione S-transferase-π (GST-π) protein was inhibited in oral squamous cell carcinoma cells dose-dependently. The expression of GST-π is positively correlated with the attenuated capacity in the cell, meaning that the higher the expression of GST-π is, the stronger the drug resistance is, and *vice versa*. In contrast to the common groups of pingyangmycin and carboplatin, drug-loaded MPEG–PLA–SS–ECA nanoparticles groups showed a more significant reduction in the expression of GST-π, suggesting that the nanoparticles coupled with GST inhibitors can effectively inhibit the expression of GST-associated proteins in the drug-resistant cells and MPEG–PLA–SS–ECA polymer materials can effectively reduce the multidrug resistance of the cells (Figure 6).

## 3. Experimental Section

### 3.1. Reagents

Pingyangmycin (Pym, Harbin Laiboten Pharmaceutical Co., Ltd, Harbin, China); carboplatin (Shandong Boyuan Co., Ltd, Jinan, China); MPEG–PLA-OH (Jinnan Daigang Biotechnology Co., Ltd, Jinan, China); Stannous octoate (Sn(Oct)2, 95%, Sigma, Carlsbad, CA, USA); methoxy polyethylene glycols (MPEG2000, Sigma, Carlsbad, CA, USA); 4-dimethylaminopyridine (DMAP, Fluka, Buchs, Switzerland); Pyrene (98%, Acros Organics, Brussels, Belgium); cysteamine hydrochloride (Acros Organics, Brussels, Belgium); carbonyl diimidazole (CDI, Sigma, Carlsbad, CA, USA); cystamine hydrochloride (Fluka, Buchs, Switzerland); dicyclohexylcarbodiimide (DCC, Sigma, Carlsbad, CA, USA); glutathione (GSH, Sigma, Carlsbad, CA, USA); *N*-hydroxysuccinimide ester (NHS, Sigma, Carlsbad, CA, USA); thiourea (Aladdin, Shanghai, China); thioglycolic acid (TGA, Aladdin, Shanghai, China); succinic anhydride (Acros Organics, Brussels, Belgium); anhydrous DMSO and anhydrous dioxane (Acros Organics, Brussels, Belgium); glacial acetic acid (analytical grade, Sinopharm Chemical Reagent Co., Ltd, Shanghai, China); dichloromethane and acetone (Sinopharm Chemical Reagent Co., Ltd, Shanghai, China); other reagents all are of analytical grade. Dialysis bag (MWCO = 3500, Nanjing Senbeijia Biological Technology Co., Ltd, Nanjing, China); silica gel plate GF254 (Shanghai Jining Industrial Co., Ltd, Shanghai, China). Thiophene (MTT, sigma, Carlsbad, CA, USA); RPMI medium 1640 (1×) liquid (GIBCO, New York, NY, USA); fetal calf serum DMEM medium (GIBCO, New York, NY, USA); thiazolyl blue tetrazolium bromide (MTT) (Shanghai Shifeng Biological Technology Co., Ltd, Shanghai, China); trypsin solution (GIBCO, New York, NY, USA); formic acid (Sinopharm Chemical Reagent Co., Ltd, Shanghai, China); Hoechst 33342 (Fluka, Buchs, Switzerland); dimethyl sulfoxide (DMSO, Sinopharm Chemical Reagent Co., Ltd, Shanghai, China); human oral carcinoma cell carcinoma SCC15 cells (provide by School of Stomatology, Jilin University, Changchun, China).

### 3.2. Synthesis of MPEG–PLA–SS–ECA Polymer

MPEG–PLA was placed in a flask, dissolved in 30 mL DCM, mixed at 0 °C, and 0.2 mol TEA was added to it; then, 0.25 mol TsCl and 12% DMAP were dissolved together in anhydrous DCM, which was dripped into the above system to react in ice bath for 6 h, and then stirred to react at room temperature overnight. It was concentrated under reduced pressure at room temperature, and then the concentrate was dissolved in 1000 mL anhydrous ether for its precipitation and suction filtration. The filtration was wetted and washed with anhydrous ether, and then dried to obtain a white dried product with a yield of 95%. The white product was dissolved in anhydrous DMSO, to which 5-fold volume of cystamine hydrochloride, 5-fold volume of TEA and 10% of DMAP were added and mixed, reacting at 38 °C. After the reaction was completed, the reaction liquid was poured into 1000 mL cold ether for the precipitation. The precipitate dissolved in 200 mL DMF was dialyzed in a dialysis bag for 24 h, which was frozen dry to get a product MPEG–PLA–NH–CH2–CH2–SS–CH2–CH2–NH2 with a yield of 76%. The lyophilized product was detected by nuclear magnetic resonance. The synthetic routes are shown in the following figure.

An appropriate amount of MPEG–PLA–NH–CH2–CH2–SS–CH2–CH2–NH2 was dissolved in anhydrous dichloromethane, and then 10% DMAP and one-time volume of ECA–CDI was added to it, which was stirred at 38 °C for 36 h. The part of the solvent volatilized by rotary evaporation was poured into cold ethyl ether for the precipitation to separate out a yellow floccule. The solution was filtrated to obtain a precipitate, and the precipitate was mixed with ethanol and ether at a volume ratio of 1:2 for its dissolution. The precipitate was dissolved in a small amount of DMF and then dialyzed, which was freeze-dried to obtain the final product, MPEG–PLA–SS–ECA, with a yield of 35%. The lyophilized product was detected by nuclear magnetic resonance.

### 3.3. Preparation of Self-Assembled MPEG–PLA–SS–ECA Nanoparticles

The drug-loaded nanoparticles were prepared by single emulsion-solvent evaporation method. Two hundred milligrams of the lyophilized MPEG–PLA–SS–ECA were dissolved in 5 mL mixed liquid of DMSO and dichloromethane for 0.5 h. After the dissolution was completed, the solution was placed in an ultrasound device, and then 30 mg of pingyangmycin and carboplatin were, respectively, dissolved in the mixed liquid of DMSO and dichloromethane, which were put in ice water bath for the ultrasound for 4 min at 450 w ultrasound strength. The organic solution was removed by rotary evaporation at a mixing speed of 300 rpm for 4 h, and the residual was centrifuged at 2000 g for 15 min to obtain nanoparticles in it and the nanoparticles were frozen dry. Pingyangmycin-, carboplatin- and nondrug- loaded MPEG–PLA–SS–ECA nanoparticles were prepared in this way, and the prepared nanoparticles were used for the subsequent tests.

### 3.4. Detection of Physicochemical Properties of MPEG–PLA–SS–ECA Nanoparticles and Study on ECA Release

The nanoparticles were observed by TEM, their apparent forms were analyzed and their particle sizes were measured with a nanoparticle size analyzer. The phosphate buffer at pH 7.4 was used as the *in vitro* drug release medium of MPEG–PLA–SS–ECA nanoparticles. Five milligrams of nanoparticles were put in a dialysis bag, and then the dialysis bag was placed in a 10 mL PBS medium and vibrated at 37 °C under a constant temperature (80 r/min) for the observation on *in vitro* release [30,31,32]. The samples were taken out at a certain interval, and 10 mL of the release medium were taken out completely and then 10 mL of the fresh medium were supplemented. The optical absorbance (OD) of release media at the maximum absorption peak at each time point was detected by UV-vis spectrophotometer, and the cumulative release percentage of ECA was calculated based on the standard curve. In order to determine the effect of GSH on the dissociation of disulfide bonds in the polymer materials, a PBS release medium containing 10 mM GSH was used for the observation. The specific procedures were the same as those described above and the cumulative release percentage of ECA was calculated based on the standard curve.

### 3.5. Study on Drug Delivery Properties of the MPEG–PLA–SS–ECA Nanoparticles

PBS release medium and PBS release medium containing 10 mM GSH were used as the delivery systems, respectively. Five milligrams of pingyangmycin- and carboplatin-loaded nanoparticles were put into dialysis bags, respectively, and then the dialysis bags were placed in a 10 mL PBS medium and vibrated at 37 °C at a constant temperature (80 r/min) for the observation on the *in vitro* release. The samples were taken out at a certain interval, and 10 mL of the release medium were taken out completely and then 10 mL of the fresh medium were supplemented. The drug release concentrations were detected by HPLC and the drug release curves were drawn [30,33]. Based on the standard curves, the cumulative release percentages were calculated, and the drug-loaded amounts of nanoparticles were calculated according to the administration dosage of drug.

### 3.6. Study on the Induction and Identification of Drug-Resistant Cells

After they were thawed and resuscitated, SCC15 cells were cultured at 5% CO_2_-saturated humidity and 37 °C in RPMI-1640 medium (including 10% calf serum, 1.5 g sodium bicarbonate, and 100 μg·mL^−^^1^ of penicillin and streptomycin). Carboplatin and pingyangmycin were used to induce the cells and the drug concentration in the culture medium was from 0.3 µg·mL^−1^; after the culture for 3 day, the medium was replaced by the normal medium and the cells were cultured in it for 2 d; when the cells grew into flakes, the drug concentration was progressively increased consecutively for 6 months until SCC15 could normally grow and passage when the mass concentration of carboplatin was maintained at 10 μg·mL^−1^ and that of pingyangmycin at 5 μg·mL^−1^, namely, the cells became drug-resistant human tongue squamous cell carcinoma cellsSCC15/CBP and SCC15/PYM.

MTT assay was used to identify the drug-resistant cells. The induced SCC15/CBP and SCC15/PYM adherent growth cells were prepared into a single cell suspension, and the cells in the single cell suspension were seeded in 96-wellplates, in which a blank control group and a negative control group were set, and 3 duplicate wells were set in each group; after the cells were cultured under conditions of 5% CO_2_ and 37 °C for 24 h, the medium was replaced by the fresh medium containing 10 μg·mL^−1^ of carboplatin and 5 μg·mL^−1^ of pingyangmycin, and then the cells were cultured again in 5% CO_2_, at 37 °C for 72 h; absorbances (*A*) of all the wells were detected at 570 nm wavelength by MTT assay for the calculation of cell survival rates.

Immunohistochemical method was used to detect the expression of P-gp in the cells and identify whether the induction of cells was successful or not. The cell growing on the glass was washed 3 times with PBS for 5 min each time, and treated with 0.25% Triton X-100 processing for 10–15 min; the extra liquid was abandoned and then the glass was washed 3 time with PBS; 3% H_2_O_2_ solution was used to block the endogenous peroxidase for 5–10 min, and then the glass was washed 3 times with PBS; mouse anti-human P-gp monoclonal antibody (1:100) was dripped on the glass at 37 °C for 2 h; the biotinylated secondary body was dripped on the glass for the incubation of the cells at 37 °C in a wet box for 0.5 h, and then the glass was washed 3 times with PBS; the biotinylated third antibody was dripped on the glass to incubate with the cells at 37 °C in a wet box for 0.5 h, and the glass was washed 3 times with PBS; the glass was stained with DAB and washed with water, then the color separation, dehydration, and transparentizing of glass were conducted, and finally the glass was observed under a microscope after it was mounted.

Growth characteristics of the drug-resistant cells were observed. The induced adherent growth cells were prepared into a single cell suspension, and the cells in the single cell suspension were seeded in 96-wellplates, in which a blank control group and a negative control group were set, and 3 duplicate wells were set in each group; the cells were cultured in 5% CO_2_ and at 37 °C for 24 h, then the medium was replaced by the fresh medium containing 10 μg·mL^−1^ carboplatin and 5 μg·mL^−1^ pingyangmycin, and the cells were cultured in 5% CO_2_ and at 37 °C for 24, 48, 72, 96, and 120 h, respectively, and finally *A* values of all the wells were detected with MTT method and the cell growth curves were calculated based on the *A* values and the culture time.

### 3.7. Study on Nanoparticle Endocytosis of the Cells

The *in vitro* uptake of MPEG–PLA–SS–ECA nanoparticle by the drug-resistant cells was observed and located under a confocal laser scanning microscope (CLSM). First the pingyangmycin was replaced by rhodamine, and then a rhodamine-labeled MPEG–PLA–SS–ECA nanoparticle was prepared in accordance with the nanoparticle preparation methods mentioned above. The induced drug-resistant oral squamous cell carcinoma cellsSCC15/PYM was seeded at a density of 10^5^/well in small confocal dishes, respectively. Twenty-four hours later, MPEG–PLA–SS–ECA and MPEG–PLA nanoparticles in solution were placed into the media containing drug-resistant oral squamous cell carcinoma cellsSCC15/PYM cells at the different time points, in which at the specified time points (0.5, 1, 2 and 4 h, respectively), 5 μg·mL^−1^ of the nanoparticles (calculated at 5 μg·mL^−1^ of frozen dry nanoparticles) were added to them; nearing 10 min of the observation, 100 μL of hochest 33342 were added to dye the nucleus of cells, then the cells were washed three times with PBS and fixed with 4% paraformaldehyde for 10 min; the fixing solution was removed and PBS was added to them, and then CLSM was used for the observation.

### 3.8. Effects of MPEG–PLA–SS–ECA Nanoparticles on MDR of the Drug-Resistant Cells

Effects of MDR MPEG–PLA–SS–ECA nanoparticles on the resistance index of drug-resistant cells were investigated. The induced inherent SCC15/CBP and SCC15/PYM cells were prepared in to a single cell suspension, and the cells were seed in 96-well plates, in which a blank control group and a negative control group were set, and 3 duplicate wells were set in each group; The cells were cultured in 5% CO_2_ and at 37 °C for 24 h, and then 0, and 5, 10, 15, 20, 25 μg·mL^−1^ mass concentrations of chemotherapeutic drugs and MPEG–PLA–SS–ECA nanoparticles containing the same mass concentrations of the chemotherapeutic drugs were added to the media in the wells, respectively; 72 h later, survival rates of the cells were detected with MTT assay. Based on the survival rate, values of the half maximal inhibitory concentration (IC_50_) were calculated, and then based on the IC_50_, the reversal fold (RF) were calculated. The formulas for the calculation are as follows: RF = IC_50_ of common drug (pingyangmycin or carboplatin)/IC_50_ of drug-loading MPEG–PLA–SS–ECA nanoparticles (pingyangmycin or carboplatin). The above experiment was repeated 3 times.

### 3.9. Detection of the Expression of GST-π Protein in Drug-Resistant Oral Squamous Cell Carcinoma Cells

Oral squamous cell carcinoma SCC15/CBP and SCC15/PYM cells growing in the logarithmic growth phase were seeded in 100 mm plates and a control group (DMSO without drug) was set; on the next day, the drug (Pingyangmycin or carboplatin) and the drug-loaded nanoparticles containing the same concentration of the drug (pingyangmycin or carboplatin) were added to the plates, which was incubated for 24 h, and then cells in the different groups were collected for the extraction of full protein; the protein concentrations in the different groups were determined and equilibrated, and after the denaturation of the protein, the samples were kept at −70 °C with cryopreservation for Western-blot testing. Forty micrograms of the protein were loaded and electrophoresed by 10% SDS-PAGE gel electrophoresis, then the electrophoretic transfer was carried out and the membrane was blocked in 5% skim milk for 2 h; the membrane was incubated with the primary antibody rabbit anti-human (GST-π (1:500); β-actin (1:1500)) antibody at 4 °C overnight, washed 3 times for 10 h with TBST, incubated with the secondary body sheep anti-rabbit (GST-π (1:2500); β-actin (1:2500)) at room temperature for 1.5 h, washed with TBST again, and the color was developed with ECL. The images were obtained, recorded and analyzed by an image Quant LAS4000 imaging system.

## 4. Conclusions

A MPEG–PLA–SS–ECA linear polymer was designed and synthesized based on principles of GST protease inhibitors and disulfide redox, and its structure was characterized by NMR method. The diameter of the self-assembly nanoparticle was 200 nm, its shape was regular and uniform, and its drug-loading was 6%. The situation of the polymer in oral squamous cell carcinoma cells was traced by a confocal microscope. The results showed that the nanoparticles could accumulate and deliver the drugs in the tumor cells. The *in vitro* release of drugs loaded by the nanoparticles showed a significant redox-sensitivity, the addition of GSH to them could accelerate the release of ECA significantly. The test for the *in vitro* cytotoxicity showed that compared with the free drug (PYM and CBP), the MPEG–PLA–SS–ECA polymer pro-drug displayed a higher cytotoxicity on the PYM- and CBP- resistant cells SCC15 and SCC15 cells, a lower IC_50_ and a redox-sensitivity. The IC_50_ of MPEG–PLA–SS–ECA/PYM and MPEG–PLA–SS–ECA/CBP nanoparticles on the drug-resistant oral squamous cell carcinoma cell lines SCC15/CBP and SCC15/PYM was 12.68 μg·mL^−1^ and 12.76 μg·mL^−1^, respectively; the RF of them in SCC15/CBP and SCC15/PYM cells was 1.51 and 1.24, respectively, indicating that MPEG–PLA–SS–ECA nanoparticles can partially reverse the resistance of these drug-resistant cells to the drugs. The results demonstrate that the synthesis of polymer by coupling with the GST-inhibitor through disulfide bonds should be successful and the polymer can reduce the multidrug resistance of tumor cells.
